# Stunned Silence: Gene Expression Programs in Human Cells Infected with Monkeypox or Vaccinia Virus

**DOI:** 10.1371/journal.pone.0015615

**Published:** 2011-01-18

**Authors:** Kathleen H. Rubins, Lisa E. Hensley, David A. Relman, Patrick O. Brown

**Affiliations:** 1 Departments of Microbiology and Immunology, Stanford University School of Medicine, Stanford, California, United States of America; 2 Department of Biochemistry, Stanford University School of Medicine, Stanford, California, United States of America; 3 United States Army Medical Research Institute of Infectious Diseases, Frederick, Maryland, United States of America; 4 Department of Medicine, Stanford University School of Medicine, Stanford, California, United States of America; 5 Veterans Affairs Palo Alto Health Care System, Palo Alto, California, United States of America; 6 Howard Hughes Medical Institute, Stanford University School of Medicine, Stanford, California, United States of America; National Institute of Allergy and Infectious Diseases, National Institutes of Health, United States of America

## Abstract

Poxviruses use an arsenal of molecular weapons to evade detection and disarm host immune responses. We used DNA microarrays to investigate the gene expression responses to infection by monkeypox virus (MPV), an emerging human pathogen, and Vaccinia virus (VAC), a widely used model and vaccine organism, in primary human macrophages, primary human fibroblasts and HeLa cells. Even as the overwhelmingly infected cells approached their demise, with extensive cytopathic changes, their gene expression programs appeared almost oblivious to poxvirus infection. Although killed (gamma-irradiated) MPV potently induced a transcriptional program characteristic of the interferon response, no such response was observed during infection with either live MPV or VAC. Moreover, while the gene expression response of infected cells to stimulation with ionomycin plus phorbol 12-myristate 13-acetate (PMA), or poly (I-C) was largely unimpaired by infection with MPV, a cluster of pro-inflammatory genes were a notable exception. Poly(I-C) induction of genes involved in alerting the innate immune system to the infectious threat, including TNF-alpha, IL-1 alpha and beta, CCL5 and IL-6, were suppressed by infection with live MPV. Thus, MPV selectively inhibits expression of genes with critical roles in cell-signaling pathways that activate innate immune responses, as part of its strategy for stealthy infection.

## Introduction

Monkeypox virus (MPV), an emerging human pathogen in the Democratic Republic of the Congo (DRC) and elsewhere in central and western Africa, produces an illness that shares clinical features with smallpox but is somewhat less lethal, with case fatality rates of approximately 10% [1], [2], [3]. An outbreak of monkeypox in the United States in 2003 demonstrated the potential for this virus to spread from traditional endemic regions [4], [5]. Despite the potential threat this virus poses to public health [3], [6], relatively little is known about the host cellular responses to MPV.

Vaccinia virus (VAC), in contrast, has been widely used as a model for understanding poxvirus biology and exploited for vaccine purposes. VAC uses a multitude of strategies to disable host immune responses [1], [7], [8], many targeting the innate immune system. Vaccinia encodes proteins that suppress the host interferon response by binding double-stranded RNA (dsRNA) and inhibiting RNA-dependent protein kinase (PKR) and 2–5 oligoadenlylate synthase (OAS) activation [9], [10], [11], [12], [13], [14], [15], an eIF2α homolog that acts as a pseudosubstrate inhibitor of PKR [16], [17], [18], proteins that serve as a decoy receptors for IFN-gamma [19], [20], [21] and IFN-αlpha/βeta [22], [23], [24], and two antagonists of host TLR signaling (A46R and A52R) [25],[26],[27]. The MPV genome encodes homologs of the VAC proteins that modulate host interferon and TLR signaling [28], [29], but the function of the putative MPV systems for suppressing of host innate immune defenses have not been examined directly. Moreover, although much is known about the activities of the VAC-encoded proteins that impair host defenses, relatively little is known about the consequences for the host transcriptional responses to this virus, and even less about other poxviruses. A few studies have characterized responses of HeLa cells to Vaccinia infection [30], [31], [32]. Yang *et al.* used deep RNA sequencing to examine the responses of HeLa cells to VAC infection and reported an increase in abundance of a small group of cellular mRNAs associated with the NF-KB pathway, inhibition of apoptosis, and signal transduction at 2 hours post infection, followed by a decrease in thousands of cellular mRNAs at 4 hours post-infection [32]. Alkhalil *et al.* described a generalized decrease in host mRNA levels 3 and 7 hours after MPV infection of *Macaca mulatta* kidney epithelial (MK2) cells. and highlighted changes in the abundance of transcripts for genes associated with ephrin signaling and actin polymerization, cell cycle progression, and the expression of ion channels [33].

To more fully investigate how these viruses alter the gene expression programs in their hosts, we characterized, with high temporal resolution, the host transcriptional programs in response to *ex vivo* infection with MPV and VAC viruses, respectively, in several different cell types, including primary human macrophages, primary human fibroblasts and HeLa cells.

## Materials and Methods

### Cells, and culture conditions

Elutriated monocytes were obtained from two different healthy adult donors or from Clonetics, Inc. (San Diego, CA), isolated according to conventional procedures, and cultured in 6 or 12 well plates containing RPMI 1640 medium and 10% heat-inactivated fetal calf serum (FCS, Invitrogen, Carlsbad, CA). The use of primary human cells for research purposes was approved by the Institutional Review Board of Stanford University. Monocytes were cultured for 2–6 days to allow for differentiation into macrophage-like cells. Primary human dermal fibroblasts were derived from autopsy skin samples after removal of keratinocytes and endothelial cells as described [34] (S100 cells) or from Clonetics Inc. (NHDF cells). S100 primary fibroblasts were cultured in Dulbecco's Modified Eagle's Medium (DMEM) with 10% FCS, glutamine, and 100 units penicillin-streptomycin (Invitrogen, Carlsbad, CA); NHDF primary fibroblasts were cultured in fibroblast growth medium 2 (Clonetics Inc.) supplemented with 2% FCS, human fibroblast growth factor-B, insulin, and gentamicin/amphotericin-B, according to the manufacturer's directions. HeLa cells (ATCC, Manassas, VA) were cultured in DMEM with 10% FCS, glutamine, and 100 units penicillin-streptomycin.

### Viruses, and infection conditions

Cells were plated in 6 or 12 well plates and incubated for 24 hours before infection with Vaccinia New York Board of Health (Wyeth, Dryvax) (VAC-NY), Vaccinia Western Reserve (VAC-WR, [35], kindly provided by B. Moss, NIH, Bethesda), or Monkeypox-Zaire (MPV-ZAI, CDC isolate V79-I-005 [28]), each at a multiplicity of infection (MOI) of 10. Control infections with Ebola-Zaire (EBOV-ZAI) [36] were also at an MOI of 10. Control killed virus infections were performed with gamma-irradiated MPV-ZAI. For preparation of killed MPV, the stock of MPV-ZAI was split into equal aliquots, half was used for the live virus infection, and half was subjected to 1×10^6^ rads of gamma-irradiation from a ^60^Cobalt source. Mock infections were performed using culture medium free of any viruses. After adsorption of virus for 1 hour at 37°C with periodic rocking, the virus-containing medium (or mock medium) was removed, cells were washed twice with phosphate-buffered saline (PBS, Invitrogen, Carlsbad, CA), and replaced with fresh culture medium. Cells were then incubated at 37°C. Pre-infection samples were collected in duplicate or triplicate for all timecourses in order to ensure a robust baseline for data analysis. Table S1 describes cells, conditions and timepoints used in these experiments. All infection timecourses were performed in a Biosafety Level 3 or 4 (if EBOV-ZAI was used) laboratory in accordance with the Biosafety in Microbiological and Biomedical Laboratories (U.S. Department of Health and Human Services) guidelines.

In some experiments, cells were first infected and then exposed to exogenous stimuli. In these experiments, primary human dermal fibroblasts or primary human macrophages (Clonetics Inc., San Diego, CA) were either mock infected (as described above), or infected with Monkeypox-Zaire at an MOI of 10. Virus was washed away and fresh medium was added. At 4 hours post-infection, phorbal 12-myristate 13-acetate (PMA) (25ng/mL final concentration , CalBiochem, La Jolla, CA), and ionomycin (1micromolar final concentration, Sigma, St. Louis, MO) [37] or poly(I-C) (100ug/mL final concentration, Sigma, St. Louis, MO) [38] were added to both the mock- and MPV-infected cultures, and serial samples were collected.

### Plaque assays

Cell supernatants were collected for virus titration and soluble factor assays at the same times that RNA was harvested; the supernatant was frozen at −80°C until subsequent analysis. Supernatants were diluted from 10−1 to 10−6 in 200 uL of MEM. Diluted supernatants were added in duplicate to confluent monolayer cultures of Vero E6 cells and incubated for 1 hour at 37°C with periodic rocking. Supernatants were removed and a 0.5% agarose solution with 2% EBME, HEPES and 10% FBS was overlayed. Cells were incubated at 37°C for 4–5 days, followed by removal of the agarose overlay. Plaques were stained with 2 mL of crystal violet solution and counted.

### Fibroblast stimulations

Primary human dermal fibroblasts were treated in 24-well plates with PBS only (mock), interferon alpha (IFNα) at 0.6 pM final concentration (Sigma, St. Louis, MO), tumor necrosis factor alpha (TNF-alpha) at 0.6 pM final concentration (Sigma), PMA at 25ng/mL final concentration plus ionomycin at 1micromolar final concentration, polyinosinic-polycytidylic acid as potassium salt (poly(I-C)) at 100ug/mL final concentration (Sigma), *Escherichia coli* 055:B5 lipopolysaccharide (LPS) at 1ug/mL final concentration (Sigma), or dexamethasone at 1 micromolar final concentration (Sigma).

### Immunofluorescence assay for cell-associated poxvirus antigen

Cells were grown on coverslips or in 4-well chamber slides and infected or mock-infected as described above, then fixed in 10% neutral buffered formalin for 24 hours, rinsed five times in PBS, permeabilized by treatment with proteinase K (20 µg/ml, DAKO, Carpinteria, CA) for 30 minutes at room temperature, followed by incubation in normal goat serum for 20 minutes (DAKO). Slides or coverslips were incubated in a 1∶1000 dilution of a mouse polyclonal anti-VAC antibody (kindly provided by B. Moss) for 30 minutes at room temperature, washed with PBS, then incubated with Alexa Fluor 488-conjugated goat anti-mouse IgG antibody (Molecular Probes, Eugene, OR) for 30 minutes at room temperature, rinsed in PBS, and mounted in an aqueous mounting medium containing 4′,6′-diamidino-2-phenylindole (DAPI) (Vector Laboratories, Burlingame, CA). Epifluorescence images were taken at 20× magnification with a Nikon E600 fluorescence microscope or Nikon Eclipse AD1 (Nikon Instech Co., Ltd., Kanagawa, Japan).

### Sample acquisition and RNA preparation for microarray analysis

Culture supernatant was collected and stored at −70°C for virus titration and soluble factor assays. Cell cultures were imaged using phase-contrast microscopy at each timepoint. Total RNA from cells was harvested at each timepoint by removing the media and adding 1mL of TriPure (Roche Applied Science, Indianapolis, IN) directly to the infected/treated cells in the culture well. RNA was extracted using TriPure reagent and linearly amplified (Ambion MessageAmp, Ambion, Austin, TX) according to the manufacturer's instructions.

### DNA microarrays and hybridization

We used human DNA microarrays containing 37,632 elements, representing 11,904 unique annotated human genes, 406 Variola and Vaccinia genes and approximately 11,000 unannotated human IMAGE cDNAs. Arrays were produced as described [39], [40]. Fluorescently-labeled cDNA prepared from amplified RNA was hybridized to the array in a two color comparative format [39], [41], with the experimental samples labeled with one fluorophore (Cy-5) and a reference pool of mRNA labeled with a second fluorophore (Cy-3). The reference pool (Universal Human Reference, Stratagene Inc.) and an equal mix of poxvirus transcripts from all timepoints provided an internal standard to enable reliable comparison of relative transcript levels in multiple samples [39], [40], [42]. Fluorescent images of hybridized microarrays were acquired using the GenePix 4000B and GenePix 4200AL microarray scanners (Axon Instruments, Union City, CA).

### Data Filtering and Analysis

Images were analyzed with GenePix Pro 5.0 (http://www.moleculardevices.com/pages/software/gn_genepix_pro.html, Molecular Devices, Sunnyvale, CA) and SpotReader (http://www.nilesscientific.com/overview.nhtml, Niles Scientific, Portola Valley, CA). Single spots or areas of the array with obvious blemishes were flagged and excluded from subsequent analyses. All non-flagged array elements were considered well-measured if the fluorescent intensity in each channel was greater than 2.5 times the local background and the regression correlation of the Cy5 and Cy3 fluorescence signal for all pixels comprising the array element was at least 0.6 for at least 80% of arrays in a dataset. Fluorescence ratios were calibrated independently for each array by applying a single scaling factor to all fluorescent ratios so that the median fluorescence ratio of array elements that passed quality-control criteria was 1.0. Data were expressed as the mean log_2_ ratio of Cy5/Cy3 fluorescence intensities, for all array elements representing a specified gene [39], [41]. Data from multiple replicates of the same day 0 sample were averaged to provide a robust “time-zero” baseline. Genes whose expression levels increased or decreased by a factor of three or more from their time-zero levels, in at least 3 of the time-course samples, were selected for further analysis. The data were stored in, and are available in their entirety from, the Stanford Microarray Database (http://smd.stanford.edu/), and GEO (accession number GSE24125) (42). All data are MIAME 2.0-compliant. For some analyses, data were hierarchically clustered using the Cluster program [43] and displayed using JavaTreeView [44].

## Results

**Figure 1:**
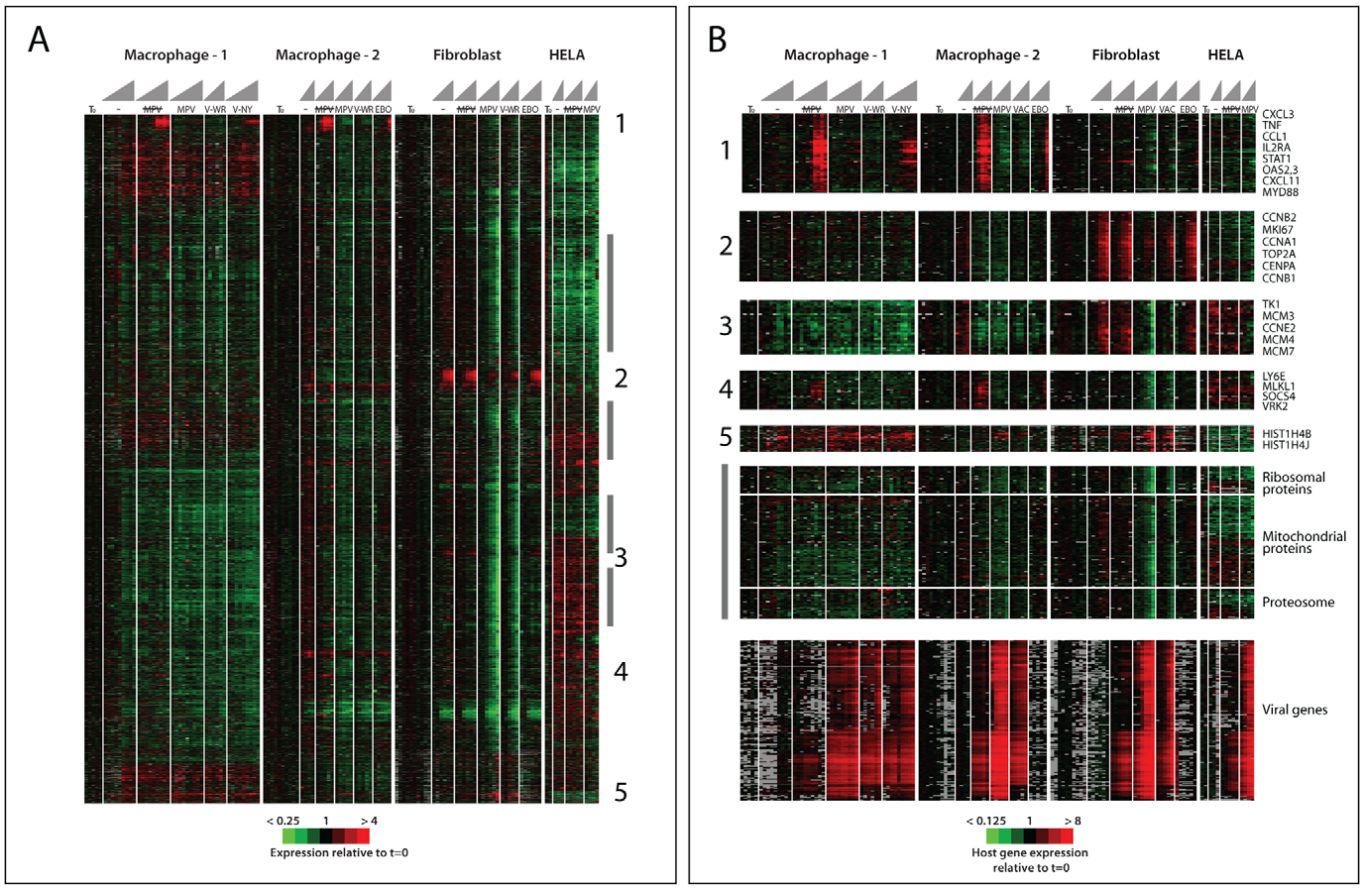
Host Gene Expression Overview. An overview of the gene expression patterns of 5745 genes whose transcript levels changed, by a factor of at least 3, from their initial levels in at least one of the 134 samples taken from time courses viral infection or mock infection in macrophages from two human donors, human dermal fibroblasts, or HELA cells. In this display, genes were hierarchically clustered based on similarities in the patterns of variation of their transcript levels [43]. In this display, each row represents a single gene, and each column represents a single cell sample from an infection or mock infection time course. The samples from each time course are ordered from left to right. Red and green colors represent expression levels greater or less, respectively, than baseline values (average of 2–5 samples taken immediately prior to infection, 0 hour time point). Grey indicates missing or excluded data. The intensity of the color reflects the magnitude of the change from baseline (Time zero), as indicated by the color scales at the bottom of each panel. Mock = mock infection (media only), V-WR = Vaccinia-Western Reserve, V-NY = Vaccinia-New York Board of Health (Wyeth, Dryvax), MPV = killed (gamma irradiated) Monkeypox-Zaire, MPV = Monkeypox-Zaire. Names of a few selected genes or categories of genes from clusters with distinctive patterns of induction or repression, are indicated to the right of enlarged views of the corresponding clusters, in panel B. Numbers to the right of panel A indicate the positions of the clusters shown enlarged and identified by the corresponding numbers in panel B. The vertical grey bars to the right of panel A indicate the positions of the genes encoding ribosomal proteins, mitochondrial proteins and proteosome components, shown in an enlarged view in panel B). For a comprehensive, browseable view of the gene expression programs depicted here, see the supporting information. The complete raw data from this experiment are available at: www.smd.stanford.edu or the GEO database (accession number GSE24125).

We infected primary human macrophages, primary human fibroblasts, and HeLa cells with Vaccinia WR and Monkeypox Zaire viruses, and as controls, a mock treatment, gamma-irradiated (killed) Monkeypox Zaire virus, and Ebola Zaire virus. We analyzed cell morphology by phase contrast microscopy (Figure S1), distribution of viral antigen by immunofluorescence staining (Figure S2), and patterns of host and viral gene expression, at high temporal resolution, using DNA microarrays (Figure 1) (The viral gene expression data were published previously [45].

### Primary human fibroblasts and HeLa cells are permissive to infection and to replication of both MPV and VAC

To assess the permissiveness of primary human fibroblasts and HeLa cells to infection with MPV and VAC, we first examined the cytopathic effects (CPE) of infection. Evidence of cell rounding was seen by 4 hours post-infection in fibroblasts and by 6 hours post-infection in HeLa cells with both MPV and VAC infection. CPE, characterized by cell rounding and aggregation, was prominent in fibroblasts by 8 hours post infection with either MPV or VAC infection (Figure S1A). By 24 hours, fibroblasts that remained adherent to the culture well formed pleomorphic cell clumps with abnormal morphology (Figure S1A). HeLa cells also showed clear CPE by 8 hours post-infection, with the majority of cells rounded by 24 hours post-infection (Figure S1B). Cells appeared more rounded after VAC infection, as compared with a multi-globular appearance after MPV infection (Figure S1B).

We evaluated the uniformity of infection by immunofluorescence with a polyclonal anti-VAC antibody that cross-reacts with both VAC and MPV antigens (B. Moss, unpublished data). MPV and VAC viral antigens, respectively, were detected in both cell types as early as 4 hours post infection (data not shown) and in every fibroblast or HeLa cell at 24-hours post-infection (Figure S2A and B). In addition, areas of viral antigen staining were detected where there were no longer viable host cells (absence of DAPI nuclear staining) (Figure S2A). Plaque assays revealed replication-competent progeny virus in the culture medium of primary human fibroblasts; 12,000 to 16,000 PFU/mL were detected at 24 hours post infection (Figure S3).

### Gene expression programs in poxvirus infected human cells

Despite the severe and pervasive cytopathic effects, the expression programs in the poxvirus-infected cells were surprisingly similar to those in the mock-infected cells. The most dramatic effect of poxvirus infection on the host transcriptome was observed in fibroblasts. Infection with live MPV or VAC led to a selective depletion, by a factor of 3 or more, of transcripts of almost 2000 genes in the primary fibroblasts, but not the macrophages or HELA cells. Another set of genes, with essential roles in cell proliferation, was induced by exposure to fresh medium in the mock-treated and killed-MPV-treated fibroblasts, but their induction was virtually abolished by live-virus infection. Importantly, the transcripts selectively depleted by poxvirus infection of fibroblasts included many of the most abundant classes of mRNAs in the cell, including those encoding ribosomal proteins, translation factors, beta and gamma actin, glycolytic enzymes, mitochondrial proteins, major components of the protein secretory apparatus, proteasome subunits, collagens, elastin and other extracellular matrix proteins. Selective depletion of this group of mRNAs could thus account for a substantial overall decrease in host mRNA during poxvirus infection of fibroblasts. No corresponding selective decrease in host mRNAs was seen in infected macrophages or HELA cells. Only a tiny set of genes actually appeared to be induced by infection with live poxviruses. Two of the 4 transcripts with the most consistent apparent induction are histone mRNAs and a third, TCF15, is also reported to be bound by SLBP [46], which allows histone mRNAs to bypass the requirement for polyadenylation. In a previous report, DNA microarray evidence for histone gene induction by Vaccinia virus, based on poly-A dependent RNA amplification, was not corroborated by Northern analysis of one of the histone mRNAs [30]. Thus, we suspect that the apparent induction of these genes by poxvirus infection may be an experimental artifact, reflecting *de novo* polyadenylation of the transcripts by the viral poly-A polymerase, greatly enhancing their amplification by the poly-A dependent procedure we used in these experiments.

### Interferon-associated gene expression

A group of about 130 genes characteristically induced by interferons, [47], [48], [49], was dramatically induced by exposure to killed MPV virus or infection with Ebola virus, but not by live MPV or VAC-WR, specifically in macrophages from two different human donors. Indeed, many of these innate immunity related transcripts actually decreased in relative abundance in response to MPV or VAC-WR infection in all three cell types (Figure 1A and B). These genes included MX1 and MX2, IP-10, OAS 1, 2, and 3, GBP 1 and 2, STAT1 and 2, CASP 1 and 10, MYD88, CXCL9, 10 and 11, CCL1, EIF2AK2 (PKR)ß, and IFN-gamma receptors 1 and 2 (IFNgR1 and 2) (Figure 1B). Interestingly, a similar infection with a different VAC strain (New York Board of Health strain, VAC-NY), did induce some of the interferon-responsive genes (Figure 1B).

### Effects of MPV infection on the transcriptional responses of human cells to diverse exogenous stimuli

**Figure 2:**
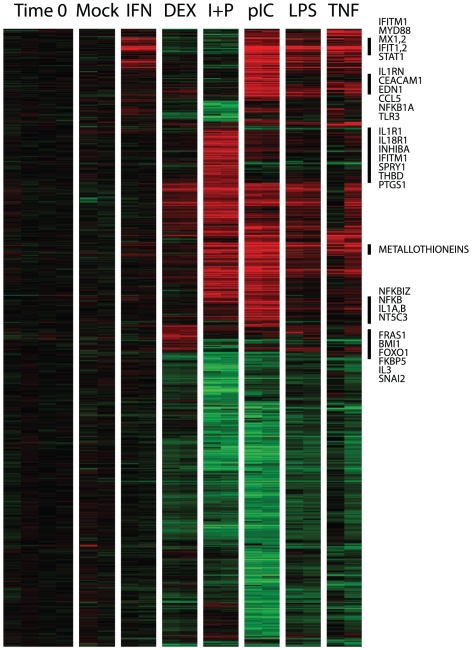
Stimulation of primary human fibroblasts. Fibroblasts were treated for 24 hours with each of 6 different molecular stimuli, or with a mock treatment. 3754 genes whose expression level increased or decreased by at least a factor of three in response to one or more of the treatments are included in this display; each column represents the changes in abundance of transcripts from each of these genes after 24 hours of treatment with one of the agents. For each molecular stimulus, results from two replicate treatments are shown. The genes were hierarchically clustered, and their expression represented by a color scale as in Figure 1. The brightest red and green colors in this display correspond to increases or decreases in transcript abundance by factors of at least 32 relative to time zero. 0hr = pre-stimulation timepoint, all other timepoints taken 24 hours post stimulation; mock (PBS), IFNα = Interferon alpha, TNF-alpha = tumor necrosis factor alpha, I+P = ionomycin and phorbal 12-myristate 13-acetate, poly(I-C) = polyinosinic-polycytidylic acid, LPS = *Escherichia coli* 055:B5 lipopolysaccharide, Dex = Dexamethasone. Names of a few selected genes from clusters with distinctive patterns of induction by one or more of these agents are indicated to the right of the corresponding clusters, marked by the vertical black lines. For a comprehensive, browseable view of the transcript profiles characteristic of each response see the supporting information. The complete raw data from this experiment are available at: www.smd.stanford.edu or the GEO database (accession number GSE24125).

We were surprised by the dramatic contrast between the major cytopathic effects following MPV or VAC infection and the relatively small extent and magnitude of the changes in transcript profiles, particularly the virtually complete absence of host transcripts induced by infection, in these mortally-infected cells. This result suggests that the viruses employed active “stealth” mechanisms to evade or silence transcriptional responses of the infected cells and minimize alarm signals that might trigger immune defenses. To identify more specifically which sensing and signaling systems might be impaired by viral infection, we first profiled the responses of primary human fibroblasts to diverse exogenous stimuli, including a “mock” treatment (PBS), interferon alpha (IFNα), Tumor necrosis factor alpha (TNF-alpha), PMA plus ionomycin (I+P) [37], [50], which mimics antigen stimulation of leukocytes, polyadenylic acid, (poly(I-C)) [38] - a double-stranded RNA mimic, *Escherichia coli* 055:B5 lipopolysaccharide [37], [51] (LPS), and dexamethasone [52], [53], each at concentrations used in previous studies. In most cases, the cells appeared normal by phase contrast microscopy 24 hours after addition of these compounds to the tissue culture media; there was cell rounding and CPE following treatment with I+P and poly(I-C) (data not shown). For each treatment, RNA was isolated from two duplicate cell culture wells after 24 hours of exposure. The fibroblasts had distinct, robust responses to these various stimuli; transcripts of more than 3600 genes increased or decreased in abundance by at least a factor of 3 in response to one or more of these stimuli (Figure 2). Some genes were induced by multiple distinct stimuli, some (e.g., CCL19, BIRC3, IL32, IFNAR2) were selectively induced by TNF, others (e.g., RARRES3,MYD88,IFIT1,IFIT2,MX1 and 2) by IFNα and still others (e.g., TGFB2, EGR1,FGF2,TLR4) by poly-IC, but not by IFNα or TNF.

**Figure 3:**
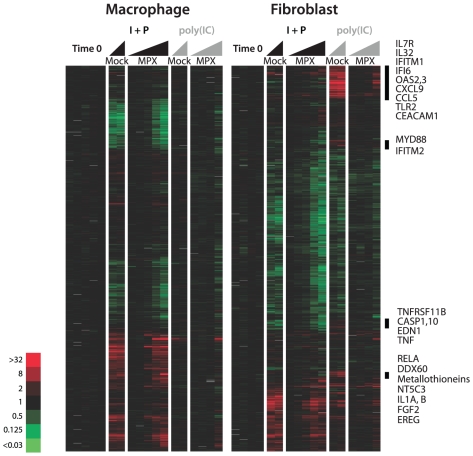
Effects of infection with MPV on the transcriptional responses of fibroblasts to poly-IC or PMA+ionomycin. (A) Fibroblasts were infected with MPV at an MOI of 10, or mock-infected, 4 hours prior to treatment with either poly(I-C) or PMA+ionomycin. Cells were harvested and RNA samples analyzed at 12 and 24 hours after treatment of mock-infected cells (16 and 28 hours after mock-infection), at 1, 2, 6 and 12 hours after poly(I-C) treatment (5, 6, 10 and 16 hours after MPV infection), and at 1, 2, 6, 12 and 24 hours after PMA and ionomycin treatment (5, 6, 10 and 16 hours after MPV infection) of MPV-infected cells. The samples from each time course are ordered from left to right. For this display, genes were clustered and expression levels represented by a color scale as in Figures 1 and 2. The brightest red and green colors in this display correspond to increases or decreases in transcript abundance by factors of at least 32 relative to time zero. Mock = mock infection (media only), MPV = Monkeypox-Zaire, I+P = treated with ionomycin and PMA 4 hours after infection or mock-infection, Poly(I-C) = treated with poly(I-C) 4 hours after infection or mock-infection. Names of a few selected genes from 4 clusters, all induced by poly(I-C) in the mock-infected cells but not in the MPV-infected cells, are indicated to the right of the corresponding clusters, marked by the vertical black lines. For a comprehensive, browseable view of the gene expression programs depicted here, see the supporting information. The complete raw data from this experiment are available at: www.smd.stanford.edu or the GEO database (accession number GSE24125).

The dramatically ablated transcriptional responses to live MPV as compared to killed MPV suggested that the live virus might actively counter one or more of the mechanisms by which these cells sense and respond to infection. To test this hypothesis, we infected primary human dermal fibroblasts and primary human macrophages with MPV, then examined their transcriptional responses to subsequent treatment with I+P or poly (I.C), beginning 4 hours post-infection. We chose I+P and poly (I.C) because these agents provoked the most pronounced responses in the uninfected fibroblasts. I+P elicited a significant response in both fibroblasts and macrophages that was virtually identical in MPV-infected and mock-infected cells (Figure 3). Poly(I-C) elicited little response in macrophages, but there was a distinct response in fibroblasts. While the responses of many genes induced by poly(I-C) in the absence of infection were essentially unimpaired by MPV infection (Figure 3), more than 100 genes induced by poly(I-C) in mock-infected fibroblasts were unresponsive to poly(I-C) following MPV infection (Figure 3). Many of these genes were known to be interferon-induced [47], [49]. Several cytokines, genes involved in vesicle transport and docking and in apoptosis were also induced by poly(I-C) in uninfected fibroblasts, but not in MPV-infected fibroblasts. Thus, the lethally infected cells were capable of robust transcriptional responses to exogenous stimuli. The failure to respond to viral infection *per se* is therefore likely to reflect not a general impairment of transcription, but active and selective viral mechanisms for inhibiting this response. In the fibroblasts, most of the critical innate-immune response genes induced by poly(IC) in uninfected cells were silent in the MPV-infected cells.

## Discussion

Viral infection involves not only extensive subversion of the host cell biosynthetic and regulatory machinery for viral replication, but also in almost all cases a “stealth” program to eliminate, mitigate or avoid responses of the innate and adaptive immune systems to the developing infection. Pox viruses have an especially elaborate and multifaceted system for silencing and evading host immune defenses. To develop a more comprehensive picture of the host cell responses to pox virus infection and viral subversion of that response, we profiled the temporal program of gene expression at high resolution during 40 time courses of infection with two different poxvirus species, in several different human cell types (Table S1). Our choice of primary human macrophages and primary human fibroblasts as the principal host cells in these studies followed from observations of smallpox and MPV infection in non-human primates, in which macrophages and fibroblasts were typically infected [54], [55]. Macrophages, in particular, have been hypothesized to be critical for the pathogenesis and spread of smallpox; as one of the initial primary target cells, circulating macrophages are believed to deliver virus to a variety of other target cells and tissues.

Poxvirus infection of these cells proceeded with extraordinary stealth. Despite unequivocal evidence of active viral infection and dramatic cytopathic changes, the gene expression programs of the host cells seemed virtually oblivious to their impending doom; in fibroblasts, many of the most abundant mRNAs were markedly depleted following poxvirus infection, but essentially none were specifically induced. Indeed, even the intense innate immune response induced by exposure of macrophages to killed MPV was virtually completely silenced in cells infected with the live virus. The apparent induction of a small group of genes by infection with either live MPV or VAC, including histone mRNAs that have previously been reported to be induced in HeLa cells by VAC WR and MVA infection, was likely an experimental artifact resulting from de novo polyadenylation of these mRNAs by the viral poly-A polymerase, leading to their enhanced amplification and detection by our microarray hybridization protocol.

A large set of interferon-responsive genes was induced by killed MPV in primary human macrophages. These genes were also induced to similar levels by an unrelated virus, Ebola-Zaire. Levels of these transcripts remained close to baseline, however, following infection with VAC WR or live MPV. The effective suppression of the transcriptional response to interferon in infected cells contrasts with the strong interferon-associated response we observed in peripheral blood mononuclear cells during *in vivo* smallpox [48] or MPV infection in non-human primates (K. Rubins, unpublished data). These contrasting results suggest that the effective suppression of the interferon response in the infected cells themselves is insufficient to quell the systemic response of a mostly uninfected population of peripheral blood cells to the consequences of overwhelming infection.

These results are broadly consistent with the predicted activities of VAC and MPV genes that encode known or putative interferon-modulating factors. In VAC, the K3L ORF functions as an eIF2α decoy homolog; the E3L ORF encodes a double-stranded RNA binding protein that inhibits 2′5′-OAS, the RNaseL system, and PKR (EIF2AK2); and the B18R and B8R ORFs are predicted to encode soluble and cell surface receptors that may act as competitive antagonists for type I and type II interferons, respectively (for review, see [8]). The expression patterns, activities and roles in pathogenesis of these interferon-modulating genes during MPV infection remain to be established. The gene that encodes the decoy eIF2α homolog is either missing or truncated in the sequenced MPV genomes [56] and the double-stranded RNA (dsRNA) binding protein is predicted only to be expressed as the short form, which, while functional for dsRNA binding and inhibiting PKR and 2′-5′ OAS activation [17], [57], does not contain an N-terminal region essential for VAC pathogenesis in a mouse model [58]. However, despite these differences, the ablation of the interferon response by poxvirus infection was quite similar in cells infected with live MPV or VAC, perhaps reflecting the ability of virally encoded secreted factors to interrupt host interferon signaling. Transcripts from the predicted dsRNA binding protein homolog in MPV as well as the Type I and II IFN decoy receptors were detected during MPV infection in these cell types. Suppression of the interferon response is not simply explained by a complete inability of virally-infected cells to respond to exogenous stimulation; in all these cells, we observed a transcriptional response to changes in the media during the initial steps of infection (Figure 1, mock versus live-virus induced transcription programs), and a vigorous response to I+P that was essentially indistinguishable from that of the uninfected cells (Figure 3). This evidence that MPV virus infection can selectively suppress the interferon response, and perhaps other components of the innate immune response, highlights the value of direct identification and investigation of the MPV genes that suppress the infected cell's ability to recruit an innate immune response.

As a foundation for defining how these viruses impaired the ability of their host cells to sense or respond to infection, we profiled the responses of the primary human fibroblast cells used in this study to chemical agents with diverse activities, including activation of innate immune responses. The fibroblasts were clearly capable of responding vigorously to these diverse stimuli. While most features of their responses to I+P or poly(I-C) appeared unaffected by MPV infection, the induction of interferon-responsive genes and several classes of genes encoding cytokines was significantly diminished in infected cells (Figure 3). In addition to suppressing the host transcriptional response to interferon in primary macrophages, MPV was able to repress a subset of genes in fibroblasts that responded specifically to poly(I-C). This repression might be due to inhibition of the TLR3 signaling pathway, which normally senses and responds to poly(I-C). The apparent blockade of TNF-alpha signaling was also interesting. Some of the genes most differentially expressed in these fibroblasts in response to TNFalpha, and not to IFNα (eg., IL7R, IL32), were induced by p(IC) in the mock-infected but not in the MPV-infected fibroblasts. Our previous work demonstrated a lack of detected circulating TNF-alpha and absence of TNF-alpha-induced transcripts in PBMCs during smallpox infection in non-human primates [48]. Poxviruses encode several homologs of the TNF-alpha receptor [59] which are believed to act as decoys. CrmB, the only TNF receptor homolog encoded by Variola that is predicted to be functional [60], is also predicted to be encoded by the J2R/J2L ORFs of MPV. The largely intact response of these fibroblasts to I+P, and the preservation of many of the transcriptional responses to poly(I-C), in the presence of MPV infection, contrasts sharply with the highly specific block to induction of IFN- and TNFa-regulated transcripts. Thus, the reprogramming of gene expression by these viruses is exquisitely specific to genes required for activation of an innate immune response.

This study represents one of the first detailed analysis of the host-cell gene expression program in response to infection with MPV, an emerging human pathogen. Remarkably, even as the infected cells were approaching their demise, their transcriptional program appeared virtually oblivious to the dramatic cytopathic effects of the overwhelming infection. Thus, the mechanisms by which poxviruses elude detection and elimination by host antiviral defenses include the silencing of cellular alarm systems that would otherwise alert the host to their presence, by selectively blocking specific transcriptional responses activated by TNF or IFNs. The specific molecular mechanisms by which these responses are blocked continue to be important questions for future investigations.

## Supporting Information

Figure S1a **Cytopathic effect (CPE) in Fibroblasts and HeLa cells infected with MPV and VAC.** Primary human dermal fibroblasts (A) or HeLa cells (B) were infected in parallel with Vaccinia Western Reserve (VAC-WR) or Monkeypox Zaire (MPV-ZAI) with 10 PFU/cell, or mock infected (Mock). Cell cultures were visualized using phase-contrast microscopy at times indicated postinfection (hrs p.i., hours post infection). Figure S1a contains complete data corresponding to Figure 1 (as calculated Data are provided as tab-delimited text in a “.cdt” format. Each row (except row 1) represents a gene and each column (except columns 1–4) represents an experimental sample. Each cell represents a quantitative measurement (on a log scale) of the ratio of the abundance of transcripts from the specified gene in the specified sample, to the same gene's abundance in the “time zero” sample (prior to infection or chemical treatment). The samples are identified by the headers in row 1. The genes are identified by name in Column 3. To open, display and browse these data with the Treeview visualization program (eg., Java Treeview [44]), rename this file as “Figure 1.cdt”, and rename Figure S1b as “Figure 1.gtr” and store them in the same folder.
(CDT)

Figure S1b This tab-delimited text file is the companion file to Figure S1a. The file contains information used to specify the dendrogram representing the relationships among expression patterns of the genes in Figure 1 as inferred by average-linkage hierarchical clustering. It is used by the “Treeview” program to generate the gene dendrogram for display and browsing. To open, display and browse the data in Figure 1 with the Treeview visualization program (eg., Java Treeview [44]), rename this file as “Figure 1.gtr”, and rename Figure S1a as “Figure 1.cdt” and store them in the same folder.
(GTR)

Figure S2a **Detection of MPV and VAC viral infection by immunofluorescence in HeLa cells and Fibroblasts.** Primary human dermal fibroblasts (A) or HeLa cells (B) were infected in parallel with Vaccinia Western Reserve (VAC-WR) or Monkeypox Zaire (MPV-ZAI) with 10 PFU/cell, or mock infected (Mock). Cells were fixed in 10% neutral buffered formalin at 24 hours post-infection. MPV or VAC viral antigen was detected with a polyclonal anti-Vaccinia antibody and a secondary antibody conjugated with Alexa 488 (green). Cell nuclei/DNA were visualized with DAPI (blue). At least 40 fields at 20× magnification each were examined for HeLa cells and fibroblasts, respectively. Figure S2a contains complete data corresponding to Figure 2 (as calculated Data are provided as tab-delimited text in a “.cdt” format. Each row (except row 1) represents a gene and each column (except columns 1–4) represents an experimental sample. Each cell represents a quantitative measurement (on a log scale) of the ratio of the abundance of transcripts from the specified gene in the specified sample, to the same gene's abundance in the “time zero” sample (prior to infection or chemical treatment). The samples are identified by the headers in row 1. The genes are identified by name in Column 3. To open, display and browse these data with the Treeview visualization program (eg., Java Treeview [44]), rename this file as “Figure 2.cdt”, and rename Figure S2b as “Figure 2.gtr” and store them in the same folder.
(CDT)

Figure S2b This tab-delimited text file is the companion file to Figure S2a. The file contains information used to specify the dendrogram representing the relationships among expression patterns of the genes in Figure 2 as inferred by average-linkage hierarchical clustering. It is used by the “Treeview” program to generate the gene dendrogram for display and browsing. To open, display and browse the data in Figure 2 with the Treeview visualization program (eg., Java Treeview [44]), rename this file as “Figure 2.gtr”, and rename Figure S2a as “Figure 2.cdt” and store them in the same folder.
(GTR)

Figure S3a **Viral growth curves following infection of human dermal fibroblasts with MPV or VAC-WR.** Supernatants collected at the indicated times after MPV (red curve) or VAC-WR (blue curve) or killed MPV (orange curve) infection of human dermal fibroblasts were serially diluted and added in duplicate to confluent monolayer cultures of Vero E6 cells. Cells were then incubated for 1 hour at 37°C with periodic rocking. The medium was then replaced with a 0.5% agarose solution with 2% EBME, HEPES and 10% FBS. Cells were then incubated at 37°C for 4–5 days, followed by removal of the agarose overlay. Plaques were stained with 2 mL of crystal violet solution and counted. The assays revealed replication-competent progeny virus in the culture medium of primary human fibroblasts following infection with MPV or VAC-WR, but not with killed MPV; 12,000 to 16,000 PFU/mL were detected at 24 hours post infection. Figure S3a contains complete data corresponding to Figure 3 (as calculated Data are provided as tab-delimited text in a “.cdt” format. Each row (except row 1) represents a gene and each column (except columns 1–4) represents an experimental sample. Each cell represents a quantitative measurement (on a log scale) of the ratio of the abundance of transcripts from the specified gene in the specified sample, to the same gene's abundance in the “time zero” sample (prior to infection or chemical treatment). The samples are identified by the headers in row 1. The genes are identified by name in Column 3. To open, display and browse these data with the Treeview visualization program (eg., Java Treeview [44]), rename this file as “Figure 3.cdt”, and rename Figure S3b as “Figure 3.gtr” and store them in the same folder.
(CDT)

Figure S3b This tab-delimited text file is the companion file to Figure S3a. The file contains information used to specify the dendrogram representing the relationships among expression patterns of the genes in Figure 1 as inferred by average-linkage hierarchical clustering. It is used by the “Treeview” program to generate the gene dendrogram for display and browsing. To open, display and browse the data in Figure 3 with the Treeview visualization program (eg., Java Treeview [44]), rename this file as “Figure 3.gtr”, and rename Figure S3a as “Figure 3.cdt” and store them in the same folder.
(GTR)

Table S1 **Cells, conditions and sampling timepoints used in these experiments.**
(PDF)

## References

[1] Esposito JJ, Fenner F, Fields BN, Knipe DM, Howley PM, Chanock RM, Melnick J (2001). Poxviruses.. Virology. 3rd ed.

[2] Heymann DL, Szczeniowski M, Esteves K (1998). Re-emergence of monkeypox in Africa: a review of the past six years.. Br Med Bull.

[3] Fenner F, Anderson DA, Arita I, Jezek Z, Ladnyi ID (1988). Smallpox and its Eradication.

[4] Reed KD, Melski JW, Graham MB, Regnery RL, Sotir MJ (2004). The detection of monkeypox in humans in the Western Hemisphere.. N Engl J Med.

[5] Di Giulio DB, Eckburg PB (2004). Human monkeypox: an emerging zoonosis.. Lancet Infect Dis.

[6] Jezek Z, Fenner F (1988).

[7] Smith GL, Symons JA, Khanna A, Vanderplasschen A, Alcami A (1997). Vaccinia virus immune evasion.. Immunol Rev.

[8] Seet BT, Johnston JB, Brunetti CR, Barrett JW, Everett H (2003). Poxviruses and immune evasion.. Annu Rev Immunol.

[9] Kim YG, Lowenhaupt K, Oh DB, Kim KK, Rich A (2004). Evidence that vaccinia virulence factor E3L binds to Z-DNA in vivo: Implications for development of a therapy for poxvirus infection.. Proc Natl Acad Sci U S A.

[10] Xiang Y, Condit RC, Vijaysri S, Jacobs B, Williams BR (2002). Blockade of interferon induction and action by the E3L double-stranded RNA binding proteins of vaccinia virus.. J Virol.

[11] Liu Y, Wolff KC, Jacobs BL, Samuel CE (2001). Vaccinia virus E3L interferon resistance protein inhibits the interferon-induced adenosine deaminase A-to-I editing activity.. Virology.

[12] Smith EJ, Marie I, Prakash A, Garcia-Sastre A, Levy DE (2001). IRF3 and IRF7 phosphorylation in virus-infected cells does not require double-stranded RNA-dependent protein kinase R or Ikappa B kinase but is blocked by Vaccinia virus E3L protein.. J Biol Chem.

[13] Sharp TV, Moonan F, Romashko A, Joshi B, Barber GN (1998). The vaccinia virus E3L gene product interacts with both the regulatory and the substrate binding regions of PKR: implications for PKR autoregulation.. Virology.

[14] Rivas C, Gil J, Melkova Z, Esteban M, Diaz-Guerra M (1998). Vaccinia virus E3L protein is an inhibitor of the interferon (i.f.n.)-induced 2–5A synthetase enzyme.. Virology.

[15] Chang HW, Watson JC, Jacobs BL (1992). The E3L gene of vaccinia virus encodes an inhibitor of the interferon-induced, double-stranded RNA-dependent protein kinase.. Proc Natl Acad Sci U S A.

[16] Davies MV, Elroy-Stein O, Jagus R, Moss B, Kaufman RJ (1992). The vaccinia virus K3L gene product potentiates translation by inhibiting double-stranded-RNA-activated protein kinase and phosphorylation of the alpha subunit of eukaryotic initiation factor 2.. J Virol.

[17] Carroll K, Elroy-Stein O, Moss B, Jagus R (1993). Recombinant vaccinia virus K3L gene product prevents activation of double-stranded RNA-dependent, initiation factor 2 alpha-specific protein kinase.. J Biol Chem.

[18] Sharp TV, Witzel JE, Jagus R (1997). Homologous regions of the alpha subunit of eukaryotic translational initiation factor 2 (eIF2alpha) and the vaccinia virus K3L gene product interact with the same domain within the dsRNA-activated protein kinase (PKR).. Eur J Biochem.

[19] Alcami A, Smith GL (1996). Soluble interferon-gamma receptors encoded by poxviruses.. Comp Immunol Microbiol Infect Dis.

[20] Mossman K, Upton C, Buller RM, McFadden G (1995). Species specificity of ectromelia virus and vaccinia virus interferon-gamma binding proteins.. Virology.

[21] Alcami A, Smith GL (1995). Vaccinia, cowpox, and camelpox viruses encode soluble gamma interferon receptors with novel broad species specificity.. J Virol.

[22] Colamonici OR, Domanski P, Sweitzer SM, Larner A, Buller RM (1995). Vaccinia virus B18R gene encodes a type I interferon-binding protein that blocks interferon alpha transmembrane signaling.. J Biol Chem.

[23] Symons JA, Alcami A, Smith GL (1995). Vaccinia virus encodes a soluble type I interferon receptor of novel structure and broad species specificity.. Cell.

[24] Alcami A, Symons JA, Smith GL (2000). The vaccinia virus soluble alpha/beta interferon (IFN) receptor binds to the cell surface and protects cells from the antiviral effects of IFN.. J Virol.

[25] Bowie A, Kiss-Toth E, Symons JA, Smith GL, Dower SK (2000). A46R and A52R from vaccinia virus are antagonists of host IL-1 and toll-like receptor signaling.. Proc Natl Acad Sci U S A.

[26] Stack J, Haga IR, Schroder M, Bartlett NW, Maloney G (2005). Vaccinia virus protein A46R targets multiple Toll-like-interleukin-1 receptor adaptors and contributes to virulence.. J Exp Med.

[27] Harte MT, Haga IR, Maloney G, Gray P, Reading PC (2003). The poxvirus protein A52R targets Toll-like receptor signaling complexes to suppress host defense.. J Exp Med.

[28] Shchelkunov SN, Totmenin AV, Babkin IV, Safronov PF, Ryazankina OI (2001). Human monkeypox and smallpox viruses: genomic comparison.. FEBS Lett.

[29] Shchelkunov SN, Totmenin AV, Safronov PF, Mikheev MV, Gutorov VV (2002). Analysis of the Monkeypox Virus Genome.. Virology.

[30] Ludwig H, Mages J, Staib C, Lehmann MH, Lang R (2005). Role of viral factor E3L in modified vaccinia virus ankara infection of human HeLa Cells: regulation of the virus life cycle and identification of differentially expressed host genes.. J Virol.

[31] Guerra S, Lopez-Fernandez LA, Conde R, Pascual-Montano A, Harshman K (2004). Microarray analysis reveals characteristic changes of host cell gene expression in response to attenuated modified vaccinia virus Ankara infection of human HeLa cells.. J Virol.

[32] Yang Z, Bruno DP, Martens CA, Porcella SF, Moss B (2010). Simultaneous high-resolution analysis of vaccinia virus and host cell transcriptomes by deep RNA sequencing.. Proc Natl Acad Sci U S A.

[33] Alkhalil A, Hammamieh R, Hardick J, Ichou MA, Jett M (2010). Gene expression profiling of monkeypox virus-infected cells reveals novel interfaces for host-virus interactions.. Virol J.

[34] Normand J, Karasek MA (1995). A method for the isolation and serial propagation of keratinocytes, endothelial cells, and fibroblasts from a single punch biopsy of human skin.. In Vitro Cell Dev Biol Anim.

[35] Smith GL, Chan YS, Howard ST (1991). Nucleotide sequence of 42 kbp of vaccinia virus strain WR from near the right inverted terminal repeat.. J Gen Virol.

[36] Jahrling PB, Geisbert J, Swearengen JR, Jaax GP, Lewis T (1996). Passive immunization of Ebola virus-infected cynomolgus monkeys with immunoglobulin from hyperimmune horses.. Arch Virol.

[37] Boldrick JC, Alizadeh AA, Diehn M, Dudoit S, Liu CL (2002). Stereotyped and specific gene expression programs in human innate immune responses to bacteria.. PNAS.

[38] Rasschaert J, Liu D, Kutlu B, Cardozo AK, Kruhoffer M (2003). Global profiling of double stranded RNA- and IFN-gamma-induced genes in rat pancreatic beta cells.. Diabetologia.

[39] Alizadeh AA, Eisen MB, Davis RE, Ma C, Lossos IS (2000). Distinct types of diffuse large B-cell lymphoma identified by gene expression profiling.. Nature.

[40] Alizadeh A, Eisen M, Davis RE, Ma C, Sabet H (1999). The lymphochip: a specialized cDNA microarray for the genomic-scale analysis of gene expression in normal and malignant lymphocytes.. Cold Spring Harb Symp Quant Biol.

[41] Eisen MB, Brown PO (1999). DNA arrays for analysis of gene expression.. Methods Enzymol.

[42] Perou CM, Sorlie T, Eisen MB, van de Rijn M, Jeffrey SS (2000). Molecular portraits of human breast tumours.. Nature.

[43] Eisen MB, Spellman PT, Brown PO, Botstein D (1998). Cluster analysis and display of genome-wide expression patterns.. Proc Natl Acad Sci U S A.

[44] Saldanha AJ (2004). Java Treeview–extensible visualization of microarray data.. Bioinformatics.

[45] Rubins KH, Hensley LE, Bell GW, Wang C, Lefkowitz EJ (2008). Comparative analysis of viral gene expression programs during poxvirus infection: a transcriptional map of the vaccinia and monkeypox genomes.. PLoS One.

[46] Townley-Tilson WH, Pendergrass SA, Marzluff WF, Whitfield ML (2006). Genome-wide analysis of mRNAs bound to the histone stem-loop binding protein.. RNA.

[47] Der SD, Zhou A, Williams BR, Silverman RH (1998). Identification of genes differentially regulated by interferon alpha, beta, or gamma using oligonucleotide arrays.. Proc Natl Acad Sci U S A.

[48] Rubins KH, Hensley LE, Jahrling PB, Whitney AR, Geisbert TW (2004). The host response to smallpox: analysis of the gene expression program in peripheral blood cells in a nonhuman primate model.. Proc Natl Acad Sci U S A.

[49] Waddell SJ, Popper SJ, Rubins KH, Griffiths MJ, Brown PO (2010). Dissecting interferon-induced transcriptional programs in human peripheral blood cells.. PLoS One.

[50] Diehn M, Alizadeh AA, Rando OJ, Liu CL, Stankunas K (2002). Genomic expression programs and the integration of the CD28 costimulatory signal in T cell activation.. Proc Natl Acad Sci U S A.

[51] Huang Q, Liu D, Majewski P, Schulte LC, Korn JM (2001). The plasticity of dendritic cell responses to pathogens and their components.. Science.

[52] Yang Y, Fang S, Jensen JP, Weissman AM, Ashwell JD (2000). Ubiquitin protein ligase activity of IAPs and their degradation in proteasomes in response to apoptotic stimuli.. Science.

[53] Wang Z, Malone MH, He H, McColl KS, Distelhorst CW (2003). Microarray analysis uncovers the induction of the proapoptotic BH3-only protein Bim in multiple models of glucocorticoid-induced apoptosis.. J Biol Chem.

[54] Jahrling PB, Hensley LE, Martinez MJ, Leduc JW, Rubins KH (2004). Exploring the potential of variola virus infection of cynomolgus macaques as a model for human smallpox.. Proc Natl Acad Sci U S A.

[55] Zaucha GM, Jahrling PB, Geisbert TW, Swearengen JR, Hensley L (2001). The pathology of experimental aerosolized monkeypox virus infection in cynomolgus monkeys (Macaca fascicularis).. Lab Invest.

[56] Weaver JR, Isaacs SN (2008). Monkeypox virus and insights into its immunomodulatory proteins.. Immunol Rev.

[57] Chang HW, Jacobs BL (1993). Identification of a conserved motif that is necessary for binding of the vaccinia virus E3L gene products to double-stranded RNA.. Virology.

[58] Brandt TA, Jacobs BL (2001). Both carboxy- and amino-terminal domains of the vaccinia virus interferon resistance gene, E3L, are required for pathogenesis in a mouse model.. J Virol.

[59] Cunnion KM (1999). Tumor necrosis factor receptors encoded by poxviruses.. Mol Genet Metab.

[60] Massung RF, Liu LI, Qi J, Knight JC, Yuran TE (1994). Analysis of the complete genome of smallpox variola major virus strain Bangladesh-1975.. Virology.

