# Supplementary material for: Stunned Silence: Gene Expression Programs in Human Cells Infected with Monkeypox or Vaccinia Virus
Source: PLoS One. 2011 Jan 18;6(1):e15615. doi: 10.1371/journal.pone.0015615 (PMC3022624; doi:10.1371/journal.pone.0015615)
Supplement: Table S1 — Cells, conditions and sampling timepoints used in these experiments. (PDF) [file pone.0015615.s007.pdf]

Cells	Source	Virus	Stimulation	Timepoints
Primary Human Macrophages	NIH, Donor 1	Mock		0, 2, 4, 12, 24
Primary Human Macrophages	NIH, Donor 1	Killed MPX		0, 2, 4, 8, 12, 24
Primary Human Macrophages	NIH, Donor 1	VAC-WR		0, 2, 4, 8, 12, 24
Primary Human Macrophages	NIH, Donor 1	MPX		0, 2, 4, 8, 12, 24
Primary Human Macrophages	NIH, Donor 1	EBOV		0, 2, 4, 8, 12, 24
Primary Human Macrophages	NIH, Donor 2	Mock		0, 0.5, 1, 1.5, 2, 4, 9, 12, 24, 48
Primary Human Macrophages	NIH, Donor 2	Killed MPX		0, 0.5, 1, 1.5, 2, 4, 9, 12, 24, 48
Primary Human Macrophages	NIH, Donor 2	VAC-NY		0, 0.5, 1, 1.5, 2, 4, 9, 12, 24, 48
Primary Human Macrophages	NIH, Donor 2	VAC-WR		0, 0.5, 1, 1.5, 2, 9, 12, 24
Primary Human Macrophages	NIH, Donor 2	MPX		0, 0.5, 1, 1.5, 2, 4, 9, 12, 24, 48
Primary Human Macrophages	Clonetics	Mock		0, 1, 2, 6, 12, 24
Primary Human Macrophages	Clonetics	Killed MPX		0, 1, 2, 6, 12, 24
Primary Human Macrophages	Clonetics	VAC-WR		0, 1, 2, 6, 12, 24
Primary Human Macrophages	Clonetics	MPX		0, 1, 2, 6, 12, 24
Primary Human Macrophages	Clonetics	Mock	I+P	0, 12, 24
Primary Human Macrophages	Clonetics	MPX	I+P	0, 1, 2, 6, 12, 24
Primary Human Macrophages	Clonetics	Mock	Poly [I.C]	0, 12, 24
Primary Human Macrophages	Clonetics	MPX	Poly [I.C]	0, 1, 2, 6, 12, 24
Primary Human Fibroblasts	Skin Biopsy	Mock		0, 1, 2, 4, 8, 12, 24
Primary Human Fibroblasts	Skin Biopsy	Killed MPX		0, 1, 2, 4, 8, 12, 24
Primary Human Fibroblasts	Skin Biopsy	VAC-WR		0, 1, 2, 4, 8, 24
Primary Human Fibroblasts	Skin Biopsy	MPX		0, 1, 2, 4, 8, 12, 24
Primary Human Fibroblasts	Skin Biopsy	EBOV		0, 1, 2, 4, 8, 12, 24
Primary Human Fibroblasts	Clonetics	Mock		0, 1, 2, 6, 12, 24
Primary Human Fibroblasts	Clonetics	Killed MPX		0, 1, 2, 6, 12, 24
Primary Human Fibroblasts	Clonetics	VAC-WR		0, 1, 2, 6, 12, 24
Primary Human Fibroblasts	Clonetics	MPX		0, 1, 2, 6, 12, 24
Primary Human Fibroblasts	Clonetics	Mock	I+P	0, 12, 24
Primary Human Fibroblasts	Clonetics	MPX	I+P	0, 1, 2, 6, 12, 24
Primary Human Fibroblasts	Clonetics	Mock	Poly [I.C]	0, 12, 24
Primary Human Fibroblasts	Clonetics	MPX	Poly [I.C]	0, 1, 2, 6, 12
HeLa cells	ATCC	Mock		0, 1, 2, 12
HeLa cells	ATCC	Killed MPX		0, 1, 2, 6, 12, 24
HeLa cells	ATCC	MPX		0, 2, 6, 12, 24
